# Zinner Syndrome: A Case Report

**DOI:** 10.7759/cureus.113033

**Published:** 2026-07-20

**Authors:** Youssef Mhamdi Alaoui, Oumayma Lahjouji, Hajar Ouazzani, Ismail Chaouche, Amal Akammar, Nizar El Bouardi, Meriem Haloua, Badreddine Alami, Moulay Youssef Alaoui Lamrani, Mustapha Maaroufi, Meryem Boubbou

**Affiliations:** 1 Mother and Child Radiology Department, Hassan II University Hospital, Sidi Mohamed Ben Abdallah University, Fez, MAR; 2 Radiology Department, Hassan II University Hospital, Sidi Mohamed Ben Abdallah University, Fez, MAR

**Keywords:** ct (computed tomography) imaging, seminal vesicle cyst, unilateral renal agenesis, ureter ectopic insertion, zinner’s syndrome

## Abstract

Zinner syndrome is an uncommon congenital disorder of the male genitourinary tract that arises from maldevelopment of the distal mesonephric (Wolffian) duct. On the affected side, it combines a seminal vesicle cyst, ejaculatory duct obstruction, and renal agenesis. Because it is frequently asymptomatic, it is often discovered incidentally on cross-sectional imaging. We report the case of a 40-year-old man under oncologic surveillance for treated colonic adenocarcinoma in whom follow-up computed tomography (CT) incidentally revealed a retrovesical cystic lesion of the left seminal vesicle associated with ipsilateral renal agenesis and ectopic insertion of the left ureter into the cyst - a constellation diagnostic of Zinner syndrome. Although magnetic resonance imaging (MRI) remains the reference standard for characterizing seminal vesicle anomalies, CT was sufficient for a confident diagnosis in this characteristic case. The absence of a directly visualized dilated ejaculatory duct did not preclude the diagnosis, as this feature is inconsistently demonstrated on CT and is usually inferred from the associated mesonephric duct anomalies. The syndrome should therefore be suspected in any man in whom a retrovesical cyst coexists with the absence of the ipsilateral kidney, and CT may confirm the diagnosis in characteristic cases even when MRI is unavailable.

## Introduction

Zinner syndrome is a rare congenital malformation of the male urogenital tract resulting from abnormal development of the mesonephric (Wolffian) duct during early embryogenesis. Its classic presentation links three abnormalities: a seminal vesicle cyst, obstruction of the ejaculatory duct, and agenesis of the kidney on the corresponding side [1,2]. Owing to its shared embryological origin in faulty mesonephric duct development, it has been regarded as the male equivalent of Mayer-Rokitansky-Küster-Hauser syndrome - a female disorder characterized by congenital aplasia or hypoplasia of the uterus and upper vagina in women with normal ovaries and a normal 46,XX karyotype, frequently associated with urinary tract anomalies [2].

Although uncommon, Zinner syndrome is increasingly recognized due to the widespread use of cross-sectional imaging. Most cases are diagnosed during the second to fourth decades of life and may remain asymptomatic or present with non-specific genitourinary symptoms [1,2].

Retrovesical cystic lesions encompass several entities that can be difficult to distinguish from one another, and recognition of the associated ipsilateral renal agenesis is the key that points to this diagnosis. We report a case of incidentally detected Zinner syndrome during routine oncologic computed tomography (CT) follow-up.

## Case presentation

A 40-year-old man with a history of surgically treated colonic adenocarcinoma, currently receiving systemic chemotherapy, was referred for contrast-enhanced thoraco-abdomino-pelvic CT as part of routine oncologic evaluation. He reported no urogenital symptoms. The relevant findings were confined to the pelvic component of the examination; the thoracic acquisition was unremarkable.

The patient had initially been admitted for large-bowel obstruction, and the emergency contrast-enhanced abdominal CT performed at that time revealed an adenocarcinoma of the right colic flexure. The left renal agenesis was reported on that examination, but the retrovesical cystic lesion was not mentioned, and the two findings were not related to each other. Surgery was confined to the right colic flexure and did not involve pelvic exploration, so the anomaly was not identified intraoperatively. It was recognized only on the present follow-up examination.

**Figure 1:**
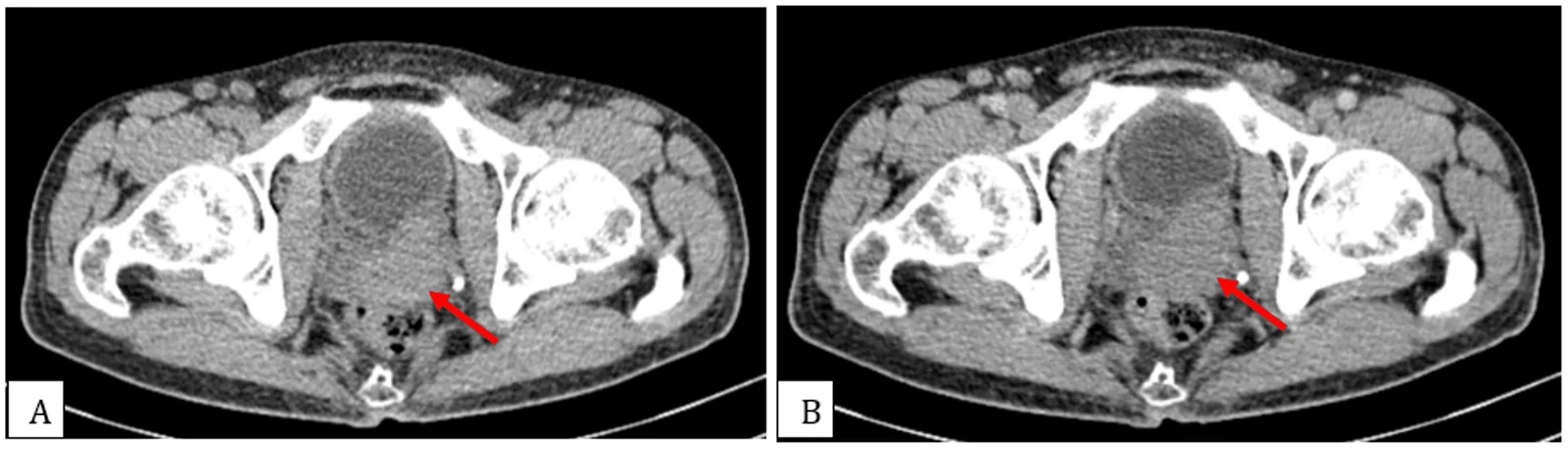
Axial computed tomography (CT) images of the pelvis. (A) Unenhanced image demonstrating a well-defined, homogeneous hyperattenuating cystic lesion (attenuation 52 HU) centered on the left seminal vesicle (red arrow). (B) Contrast-enhanced image showing unchanged attenuation (52 HU; ΔHU = 0) with no appreciable enhancement of the lesion (red arrow), confirming its non-solid nature. HU: Hounsfield units

On the pelvic images, a well-circumscribed cystic lesion arising from the left seminal vesicle was identified, measuring 42 × 39 mm in axial dimensions. On unenhanced images, the lesion showed homogeneous spontaneous hyperattenuation, with a region-of-interest attenuation of 52 HU - well above that expected for simple fluid and clearly distinct from the negative attenuation of fat - suggesting proteinaceous or hemorrhagic content. No internal septations, mural nodules, or calcifications were present. After intravenous contrast administration, the attenuation was unchanged (52 HU; ΔHU = 0), confirming the absence of enhancement and the non-solid nature of the lesion (Figure 1).

**Figure 2:**
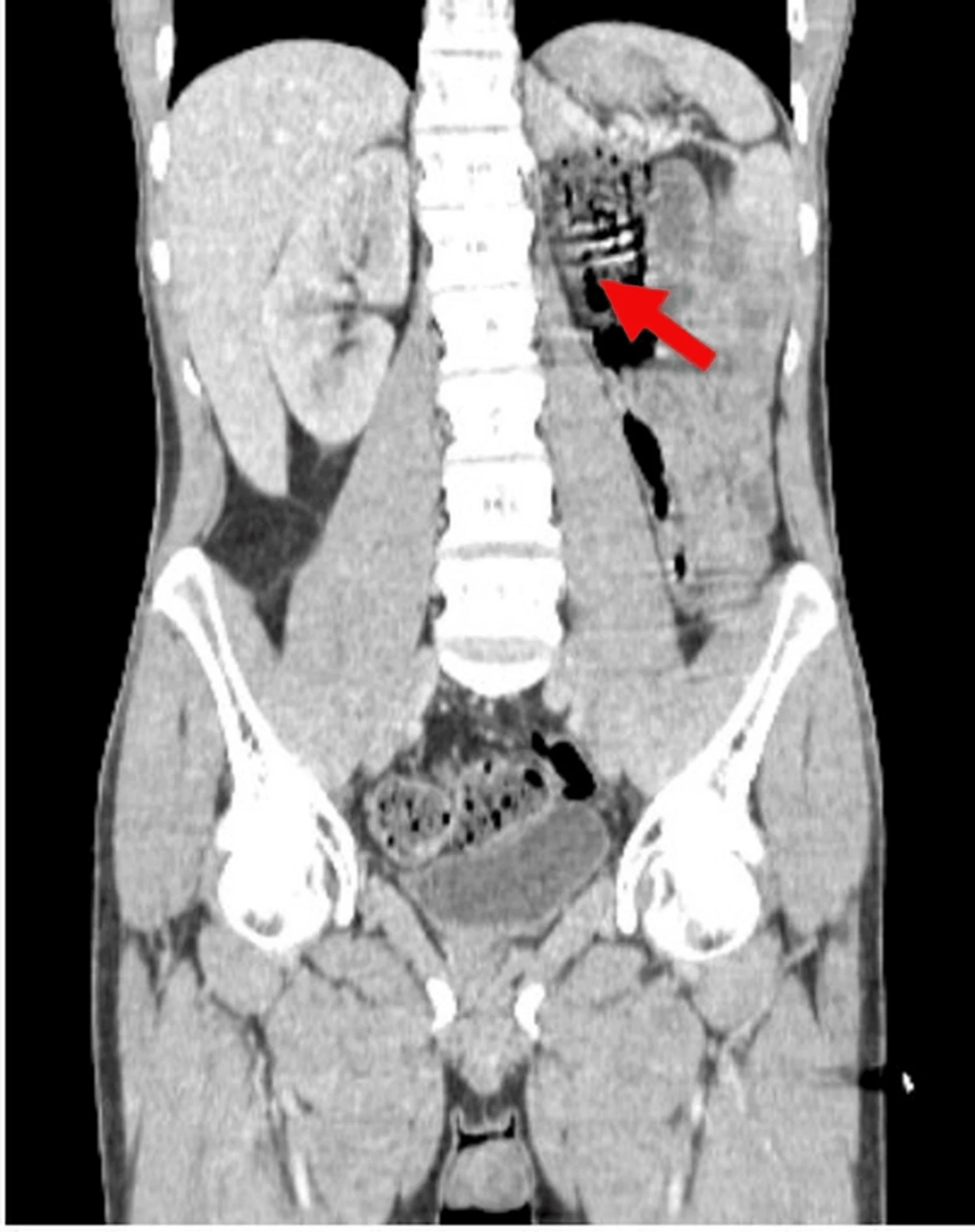
Coronal contrast-enhanced computed tomography (CT) image of the abdomen and pelvis demonstrating complete absence of the left kidney in the renal fossa (red arrow), with no evidence of ectopic renal tissue, consistent with ipsilateral renal agenesis.

**Figure 3:**
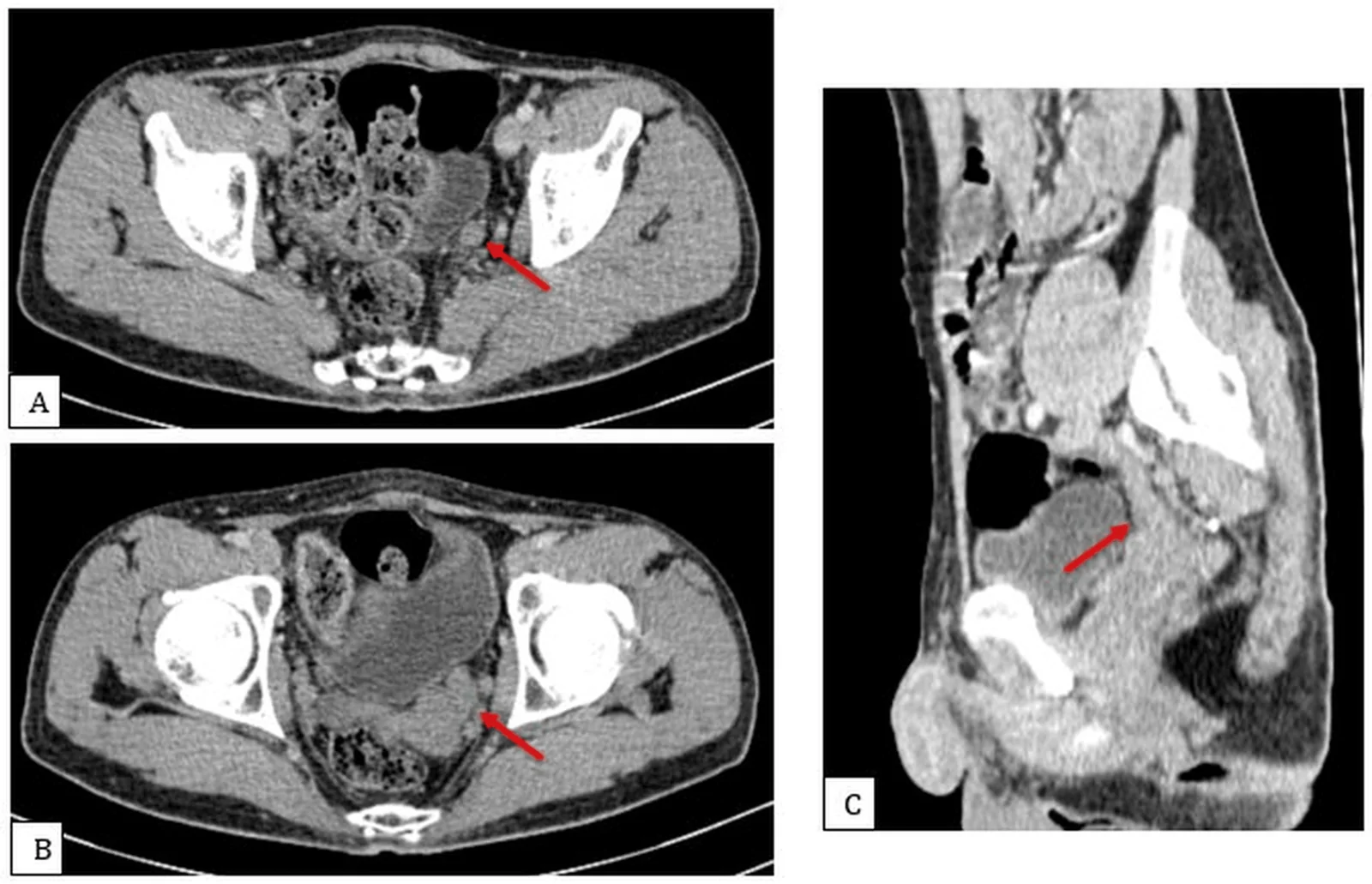
Contrast-enhanced computed tomography (CT) images. (A) Axial image of the pelvis demonstrating the left ureter (red arrow). (B) Axial image and (C) oblique sagittal reconstruction showing ectopic insertion of the left ureter into the seminal vesicle cystic lesion (red arrow).

Further evaluation revealed a complete absence of the left kidney in the renal fossa, with no evidence of ectopic renal tissue (Figure 2). A tubular structure consistent with the left ureter was identified and could be traced distally to an ectopic insertion into the seminal vesicle cystic lesion (Figure 3). The right kidney and contralateral urinary tract were unremarkable.

Based on the association of a seminal vesicle cyst, ipsilateral renal agenesis, and ectopic ureteral insertion, a diagnosis consistent with Zinner syndrome was established.

## Discussion

The findings in our patient are characteristic of the syndrome. Although it is classically described as a triad (ipsilateral renal agenesis, seminal vesicle cyst, and ejaculatory duct obstruction), it is more accurately regarded as part of a broader spectrum of mesonephric duct anomalies, since not every component is consistently demonstrable on imaging [2,3].

Embryologically, the ureteric bud arises from the mesonephric duct and interacts with the metanephric blastema to form the definitive kidney. Failure of this process leads to renal agenesis, while concomitant abnormalities of the distal mesonephric duct result in ejaculatory duct obstruction and cystic dilatation of the seminal vesicle [1]. The presence of an ectopic ureter draining into the seminal vesicle, as observed in our case, reflects incomplete separation of the ureteric bud and further supports this embryological mechanism [2].

Clinically, Zinner syndrome is often diagnosed during the second to fourth decades of life, although many patients remain asymptomatic. When present, symptoms are non-specific and may include dysuria, perineal pain, urinary frequency, painful ejaculation, or infertility [2]. Small cysts are frequently asymptomatic, explaining incidental discovery in many cases [1].

Imaging plays a central role in diagnosis. Ultrasound may detect a cystic pelvic lesion, but cross-sectional imaging is essential for accurate characterization. CT typically demonstrates a well-defined retrovesical cystic lesion associated with ipsilateral renal agenesis [4]. However, magnetic resonance imaging (MRI) is considered the gold standard due to its superior soft-tissue resolution and ability to confirm the seminal vesicle origin and characterize cyst content [3,5].

Although MRI is widely regarded as the imaging modality of choice, it was not performed in our case, as the diagnosis was established on contrast-enhanced CT obtained in the context of oncologic follow-up. The CT findings were sufficiently characteristic, demonstrating the association of a seminal vesicle cyst, renal agenesis, and ectopic ureteral insertion, allowing confident diagnosis without further imaging. We regard this as a situation in which CT was sufficient in a characteristic case, rather than a general substitute for MRI, which retains clear advantages when the origin of the lesion or its content remains uncertain.

The spontaneous hyperattenuation measured in our case (52 HU, unchanged after contrast) indicates proteinaceous or hemorrhagic content and objectively confirms the absence of enhancement; the stable attenuation excludes an enhancing soft-tissue lesion, and the measured value - well above that of simple fluid and far from the negative attenuation of fat - is consistent with a complicated cyst. This appearance is a recognized but under-emphasized pitfall, because a hyperdense pelvic lesion in an oncologic patient may otherwise raise concern for metastatic disease or a complicated cystic neoplasm [1].

In our patient, the dilated ejaculatory duct was not directly visualized. Its obstruction is therefore inferred from the associated mesonephric duct anomalies rather than demonstrated, so the diagnosis is best characterized as a presumptive, imaging-consistent manifestation of the syndrome. This limitation is common on CT and does not preclude the diagnosis when the remaining features are characteristic.

Our observation is concordant with previously reported cases, including series in which the diagnosis rested on CT alone and reports emphasizing the ectopic ureteral insertion as a supportive embryological sign [1,2]. The demonstration of the ectopic ureter draining into the cyst, which is not always documented in the literature, reinforces the diagnostic reasoning in the present case.

The differential diagnosis of retrovesical cystic lesions includes Müllerian duct cysts, prostatic utricle cysts, ejaculatory duct cysts, ectopic ureterocele, and cystic neoplasms. The decisive feature of Zinner syndrome is its association with ipsilateral renal agenesis; in addition, a strictly midline location favors Müllerian or utricle cysts, whereas a paramedian, seminal-vesicle-centered location supports the diagnosis (Table 1) [2,5].

**Table 1:** Differential diagnosis of retrovesical cystic lesions and the features that distinguish Zinner syndrome. HU: Hounsfield units

Entity	Typical location	Key associated feature	Main distinguishing point
Seminal vesicle cyst (Zinner syndrome)	Paramedian, arising from the seminal vesicle	Ipsilateral renal agenesis; ectopic ureter may drain into the cyst	The associated ipsilateral renal agenesis is the decisive diagnostic clue
Müllerian duct cyst	Strictly midline, posterior to the bladder base	Kidneys typically normal	Midline location; may extend above the prostate; no renal agenesis
Prostatic utricle cyst	Midline, within or at the prostate	May be associated with hypospadias and, in some cases, ipsilateral renal agenesis	Small, intraprostatic; communicates with the prostatic urethra
Ejaculatory duct cyst	Paramedian, along the course of the ejaculatory duct	May cause obstruction or infertility	Small, follows the duct; not associated with renal agenesis
Ectopic ureterocele	At the bladder base/trigone	Duplicated collecting system	Contains urine, continuous with a ureter, opacifies on delayed phases
Cystic neoplasm (e.g., cystadenoma)	Variable	Solid components, thick septa, or growth over time	Enhancing solid/septal elements; no renal agenesis

Management depends on symptomatology and cyst size. Asymptomatic patients are typically managed conservatively with clinical and imaging follow-up, while surgical intervention is reserved for symptomatic cases, large cysts, or infertility [2]. Accordingly, in our asymptomatic patient, no intervention was undertaken, and the anomaly was followed within his ongoing oncologic surveillance.

This case highlights the importance of recognizing Zinner syndrome as a potential incidental finding on CT, particularly in oncologic settings. Awareness of this entity is essential to avoid misinterpretation and unnecessary diagnostic or therapeutic interventions.

## Conclusions

Zinner syndrome is a rare but characteristic congenital anomaly that should be considered whenever a retrovesical cystic lesion is associated with ipsilateral renal agenesis. When this association is unequivocal, CT can be sufficient for a confident diagnosis, although MRI remains the reference standard for detailed characterization and should be obtained when the origin or content of the lesion is uncertain. As observations drawn from a single case, these points are primarily of educational value; their main practical message is the importance of recognizing congenital genitourinary anomalies during oncologic imaging in order to prevent misinterpretation as pelvic malignancy.
